# Knowledge, attitudes, risk perception of influenza and influenza vaccination among final year nursing students in Singapore: an exploratory study

**DOI:** 10.1186/2047-2994-4-S1-P19

**Published:** 2015-06-16

**Authors:** R Leong

**Affiliations:** 1 Nursing, JurongHealth, Singapore

## Introduction

In Singapore, Influenza causes about 600 deaths (out of 15,000) affecting people who are over 65 years old ('Ministry of Health: FAQs', 2014). Influenza vaccination benefits health-care workers (HCWs) and reduces influenza-related morbidity and mortality in high-risk patients. However, only 25% of student nurses have reported having received seasonal or H1N1 vaccination [1].

## Objectives

To explore the relationship among final year nursing students’ knowledge, risk perception, health beliefs and their influenza vaccination behaviours and uptake in Singapore.

## Study design

*This* study utilised a non-experimental, cross-sectional exploratory quantitative study design.

## Methods

Convenience sampling was used to recruit 868 final year nursing students in Singapore from October 2013 to January 2014. Two survey forms were used to collect data: (i) Participants’ Demographic Information sheet and (ii) King’s Nurses Influenza Vaccination Questionnaire (KNIVQ).

## Results

Student nurses’ vaccination rates were 15.7% for Seasonal Influenza vaccine and 5.4% for Influenza A (H1N1) vaccine. Findings revealed a relationship between vaccination uptake rates and knowledge and risk perception and student nurses’ perceived health locus of control (p<0.05). The results also described the different level of student nurses’ vaccination behaviours and their vaccination recommendation to patients. Vaccinated student nurses were also more likely to recommend the Influenza vaccine to their patients in the future.

## Conclusion

The findings provide valuable input enabling policy makers develop measures to improve influenza vaccination awareness and take-up rates among students, encouraging them to be patient advocates regarding importance of influenza vaccination for health prevention and promotion.

## Disclosure of interest

None declared.
